# Evolution of meiotic recombination genes in maize and teosinte

**DOI:** 10.1186/s12864-017-3486-z

**Published:** 2017-01-25

**Authors:** Gaganpreet K. Sidhu, Tomasz Warzecha, Wojciech P. Pawlowski

**Affiliations:** 1 School of Integrative Plant Science, Cornell University, Ithaca, NY 14853 USA; 2 Current address: Institute for Cancer Genetics, Columbia University, New York, NY 10032 USA; 3 Permanent address: Department of Plant Breeding and Seed Science, Agricultural University, Krakow, Poland

**Keywords:** Genetic variation, Recombination, Meiosis, Molecular evolution, Domestication

## Abstract

**Background:**

Meiotic recombination is a major source of genetic variation in eukaryotes. The role of recombination in evolution is recognized but little is known about how evolutionary forces affect the recombination pathway itself. Although the recombination pathway is fundamentally conserved across different species, genetic variation in recombination components and outcomes has been observed. Theoretical predictions and empirical studies suggest that changes in the recombination pathway are likely to provide adaptive abilities to populations experiencing directional or strong selection pressures, such as those occurring during species domestication. We hypothesized that adaptive changes in recombination may be associated with adaptive evolution patterns of genes involved in meiotic recombination.

**Results:**

To examine how maize evolution and domestication affected meiotic recombination genes, we studied patterns of sequence polymorphism and divergence in eleven genes controlling key steps in the meiotic recombination pathway in a diverse set of maize inbred lines and several accessions of teosinte, the wild ancestor of maize. We discovered that, even though the recombination genes generally exhibited high sequence conservation expected in a pathway controlling a key cellular process, they showed substantial levels and diverse patterns of sequence polymorphism. Among others, we found differences in sequence polymorphism patterns between tropical and temperate maize germplasms. Several recombination genes displayed patterns of polymorphism indicative of adaptive evolution.

**Conclusions:**

Despite their ancient origin and overall sequence conservation, meiotic recombination genes can exhibit extensive and complex patterns of molecular evolution. Changes in these genes could affect the functioning of the recombination pathway, and may have contributed to the successful domestication of maize and its expansion to new cultivation areas.

**Electronic supplementary material:**

The online version of this article (doi:10.1186/s12864-017-3486-z) contains supplementary material, which is available to authorized users.

## Background

Meiotic recombination produces genetic variation by creating new combinations of alleles, and facilitates purging of deleterious mutations from genomes and populations [1]. While the role of recombination in evolution is well recognized, the evolution of the recombination pathway itself has received little attention. Components of the recombination pathway exhibit a high degree of overall conservation across eukaryotes [2], suggesting a preponderance of purifying selection during evolution. Nevertheless, frequencies of recombination events and their distribution across the genome differ between as well as within species [3–9]. Differences also exist between species in the presence or absence of certain recombination pathway components [2, 10]. These observations suggest that at least some recombination genes are under more flexible evolutionary constraints. Indeed, patterns of sequence polymorphisms indicative of positive selection have been found in meiotic and recombination-related genes in *Drosophila melanogaster* and *D. simulans* [11], as well as diploid and tetraploid *Arabidopsis arenosa* [12–14]. Furthermore, it has been proposed that environmental as well as genomic changes, such as whole-genome duplication, are triggers of adaptive changes in meiosis and meiotic recombination [15].

**Fig. 1:**
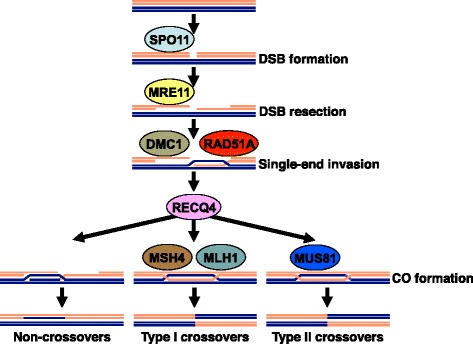
The main steps of the meiotic recombination pathway in maize. Only the proteins investigated in this study are shown. Each chromosome is of a different color. For simplicity, only one chromatid is shown for each chromosome

To gain more insight into the patterns of recombination gene evolution, we studied sequence polymorphism in genes involved in key steps of the meiotic recombination pathway in maize and teosinte. Modern maize is a product of a single domestication event from Balsas teosinte (*Zea mays* ssp. *parviglumis*) that occurred about 8700 years ago in the Balsas River valley in southern Mexico [16, 17]. Maize has maintained a substantial proportion (60–70%) of the genetic variation found in *Z. mays* ssp. *parviglumis*, and is more diverse than its more distantly related wild relative *Z. luxurians* [18–21]. However, during domestication and subsequent breeding, a small fraction of maize genes (“domestication genes”) have been subject to very strong selection pressures and have lost most if not all of their pre-domestication diversity [19, 20, 22, 23]. Theoretical predictions [24] as well as empirical studies [25] indicate that populations experiencing directional or strong selection pressures are likely to evolve increased recombination rates. Higher recombination rates should be of most value when selection is strong and genetic variability is limited [26], such as during domestication. Indeed, increases in meiotic recombination rates have been shown to accompany domestication of several plant species, including maize [27]. Selection to alter recombination patterns might leave footprints in sequences of recombination genes. To examine this issue, we analyzed patterns of sequence polymorphisms in several genes controlling key steps of meiotic recombination in maize (Fig. 1).

Meiotic recombination is initiated by formation of double-strand breaks (DSBs) in chromosomal DNA by SPO11, a protein belonging to the topoisomerase family [28–30]. The DSBs are subsequently resected from 5′ to 3′ by a protein complex known in plants and animals as MRN to generate single-stranded DNA (ssDNA) overhangs [31, 32]. MRE11 is a key component of this complex. It possesses endonuclease, exonuclease, and helicase activities that directly facilitate the ssDNA overhang formation [32, 33]. The ssDNA ends are coated by two DNA strand-exchange proteins, RAD51A and DMC1, and invade homologous double-stranded DNA regions [34]. Eventually, meiotic DSBs are repaired into either crossovers (COs) or non-crossovers (NCOs). A RecQ helicase SGS1, is one of the best studied of several regulators thought to control the CO/NCO decision [35]. The functional homolog of SGS1 in plants is RECQ4 [36, 37]. CO formation is, furthermore, regulated by a phenomenon of CO interference, which prevents formation of multiple COs in the same chromosome region [38, 39]. In plants, as well as yeast and mammals, there are two CO types, type I COs that are subject to interference, and type II COs that are not [39–44]. The two CO classes are outcomes of parallel pathways, which are facilitated by different complexes of recombination proteins. MSH4 and MLH1 act in formation of type I COs [42, 45], while MUS81 is involved in type II CO formation [43, 46].

Overall, we examined sequence polymorphisms in eleven recombination genes in maize and teosinte. We found that, despite their overall conservation, most of the recombination genes exhibited detectable patterns of sequence evolution. Several genes showed polymorphism patterns indicative of adaptive evolution.

## Results

### Genomic organization of maize meiotic recombination genes

To study patterns of sequence polymorphism in meiotic recombination genes, we selected genes encoding eight proteins that facilitate key steps of the recombination pathway (Fig. 1): DMC1, MLH1, MRE11, MSH4, MUS81, RAD51A, and SPO11. As the first step of the study, we investigated the presence and organization of these genes in the maize genome. MRE11 and RAD51A were studied in maize before and examined at the functional level [31, 47–49]. These studies showed that the maize genome contains two homologs of *Mre11* (*Mre11A* and *Mre11B*) and *Rad51A* (*Rad51A1* and *Rad51A2*). We confirmed these findings (Additional file 1: Table S1) using the complete draft of the maize genome sequence [50]. DMC1, MLH1, MSH4, MUS81, RECQ4, and SPO11 have not yet been studied in maize at the functional level but they have been examined in Arabidopsis [29, 30, 36, 37, 42, 43, 45, 46, 51–53]. We identified maize homologs of the genes encoding these six proteins by searching maize genomic and EST sequence resources using TBLASTN with Arabidopsis protein sequences as queries. In Arabidopsis, DMC1, MLH1, MSH4, MUS81 are encoded by single genes. Analysis of the maize genome sequence revealed that *Dmc1*, *Mlh1*, and *Msh4* were also present as single full-length genes in maize (Additional file 1: Table S1). In contrast, we found two sequence homologs of *Mus81*. These two genes, *Mus81-1* and *Mus81-2*, shared a rather limited 46% identity and 62% similarity at the amino acid level.

The Arabidopsis genome contains two closely related copies of *RECQ4*, *RECQ4A* and *RECQ4B* [52, 54], which are products of a fairly recent duplication event [54]. In contrast, maize, as well as most other plant species, have single *RECQ4* homologs (Additional file 1: Figure S1).

SPO11 in Arabidopsis is represented by three isoforms thought to stem from an ancient gene duplication in now extinct basal eukaryotes [55, 56]. However, only two of the three *SPO11* genes, *SPO11-1* and *SPO11-2*, function in meiotic recombination [29, 30]. The maize genome contains single homologs of all three Arabidopsis *SPO11* genes.

### Origin of the duplicated recombination genes in maize

**Fig. 2:**
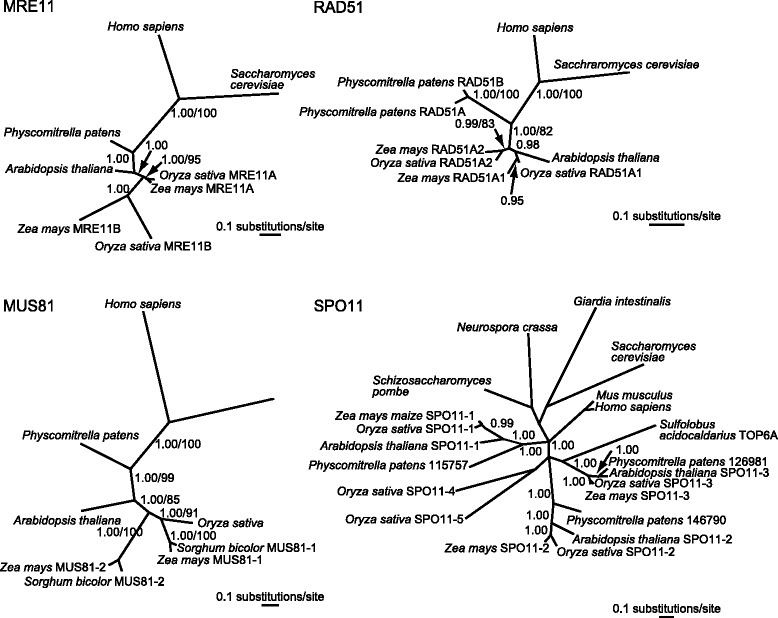
Phylogeny reconstructions of the MRE11, MUS81, RAD51, and SPO11 proteins in eukaryotes based on the Bayesian and maximum parsimony methods. For all proteins, except SPO11, the Bayesian trees and the maximum parsimony trees were identical. For SPO11, the Bayesian tree is shown. Numbers next to branches are posterior probabilities and bootstrap support values. Only posterior probability values of 0.8 and higher and bootstrap support values of 70% and higher are reported. Accession numbers for sequences from GenBank used in this analysis are listed in Additional file 1: Table S2

To examine if presence of duplicated copies of *Mre11*, *Mus81-1*, *Rad51A*, and *Spo11* was unique to maize in the context of homologs of these genes present in other eukaryotes, we conducted phylogenetic analyses of amino acid sequences from several representative species using Bayesian as well as maximum parsimony (MP) methods (Fig. 2 and Additional file 1: Figure S1). Both methods produced essentially identical trees, except for SPO11, where the Bayesian tree provided a finer resolution of the phylogenetic relationships than the MP tree.

The maize genome is thought to be a product of an allopolyploidization event that occurred about 5 to 12 Mya between two ancestors that diverged from each other about 12 Mya [57–59]. Franklin et al. proposed that the presence of two *Rad51A* homologs in maize is a result of this duplication [48]. However, phylogeny reconstructions revealed that *Rad51A*, as well as *Mre11*, became duplicated before the divergence of the maize and rice lineages (Fig. 2). *Mus81* also has undergone a duplication before the maize-rice divergence but in the rice lineage the copy corresponding to maize *Mus81-2* was subsequently lost. According to Tajima’s 1D relative rate test, maize *Mus81-2* had a much accelerated evolution rate compared to *Mus81-1* (*P* = 0.00465; Arabidopsis was used as outgroup). This finding created uncertainty whether *Mus81-2* retained the same function in meiosis as *Mus81-1*. The *Mus81-1* gene has been shown to play a role in recombination in rice [60]. In contrast, no functional information exists for *Mus81-2* in any species. Consequently, we decided to only use *Mus81-1* in further analyses. Interestingly, Hartung et al. [54] also identified a second *MUS81*-like gene in Arabidopsis (At5g39770) but were unable to amplify any, even partial, cDNA using primers to different regions of the predicted mRNA, leading them to conclude that this sequence represented a non-functional pseudogene.

Reconstruction of the *Spo11* phylogeny (Fig. 2) revealed that the three *Spo11* genes present in maize, *Spo11-1*, *Spo11-2*, and *Spo11-3*, are orthologs of the three Arabidopsis *SPO11* genes. Interestingly, the rice genome contains two additional homologs of *SPO11*, *SPO11-4* and *SPO11-5*, which are not present in maize, sorghum, or any other plant species with a sequenced genome, and have been so far only identified in *japonica* and *indica* rice [61, 62]. These data suggest that both *SPO11-4* and *SPO11-5* originated after the maize-rice divergence. However, they do not appear to belong to either of the plant SPO11-1, SPO11-2, or SPO11-3 clades, so their exact origin is not clear.

### Sequence diversity in maize recombination genes

As the first step to characterize polymorphism patterns in the recombination genes, we examined their sequence diversity in maize and teosintes. To do this, we sequenced eleven genes, *Dmc1*, *Mlh1*, *Mre11A*, *Mre11B*, *Msh4*, *Mus81-1*, *Rad51A1*, *Rad51A2*, *Recq4*, *Spo11-1*, and *Spo11-2*, from a set of 31 diverse maize inbred lines. The inbreds were selected to maximize genetic diversity [63] and included 25 of the 26 founders of the Nested Association Mapping (NAM) population, representing more than 80% of the allelic diversity of maize [64]. The 31 inbreds included both tropical (A188, A344, CML52, CML69, CML103, CML228, CML247, CML277, CML322, CML333, Ki3, Ki11, M37W, Mo18w, NC350, NC358, and Tx303) and temperate (B73, B97, CO106, CO125, CO255, HP301, Il14h, Ky21, M162w, Mo17, MS71, Oh7b, Oh43, and P39) lines. We also used nine lines of *Zea mays* ssp. *parviglumis* (Balsas teosinte), the direct wild ancestor of cultivated maize [16, 17]. In addition, we included several more-distantly related teosinte accessions representing *Z. mays* ssp. *mexicana*, *Z. mays* ssp. *huehuetenangensis*, *Z. diploperennis*, and *Z. luxurians*. For each gene, we sequenced the entire coding region (Additional file 1: Table S1), up to 240 bp of the region upstream from the ATG codon, and between 826 and 3605 bp of intron fragments.

To characterize sequence diversity of the recombination genes, we focused on their coding regions, as our interest was to study sequence diversity patterns that may affect protein function. We examined nucleotide sequence diversity by calculating two commonly used diversity statistics: *π*, the average number of nucleotide differences per site between any two sequences in sample [65], and *θ*_*W*_, which is a scaled measure of the number of polymorphic nucleotide sites per nucleotide [66]. The calculations were conducted on a set of 25 maize inbreds that were in common for the eleven gene datasets. The nucleotide diversity estimates varied more than seven-fold among the genes (Table 1). Furthermore, we detected larger-than-four-fold differences between paralogs in the three duplicated genes, *Mre11*, *Rad51A*, and *Spo11*.

**Table 1:** Nucleotide and amino acid sequence diversity and nucleotide sequence divergence in coding regions of recombination genes in a common set of 25 maize inbred lines

Gene	Alignment length (bp)	Diversity						Divergence	
		Nucleotide diversity in the coding region					Amino acid diversity (polymorphic amino acid residues per 100 residues)	*d*_N_/*d*_S_^a^	K scale factor^b^
		*θ*_*W*_	*π* (+/−SE)	*π*_*a*_	*π*_*s*_	*π*_*a*_/*π*_*s*_			
*Dmc1*	1032	0.00436	0.00332 +/− 0.000104	0.00040	0.01269	0.032	0.87	0.013	1.74
*Mlh1*	1197	0.00310	0.00229 +/− 0.000078	0.00113	0.00600	0.188	1.66	0.157	0.43
*Mre11A*	2124	0.00288	0.00229 +/− 0.000048	0.00036	0.00886	0.041	0.85	0.014	1.20
*Mre11B*	2019	0.00224	0.00123 +/− 0.000056	0.00112	0.00162	0.691	2.23	0.370	1.00
*Msh4*	2412	0.00231	0.00321 +/− 0.000058	0.00088	0.01054	0.083	0.62	0.074	0.82
*Mus81-1*	1089	0.00244	0.00217 +/− 0.000062	0.00182	0.00361	0.504	2.00	0.129	0.76
*Rad51A1*	873	0.00212	0.00162 +/− 0.000084	0.00000	0.00832	0.000	0.00	0.000	2.18
*Rad51A2*	666	0.00398	0.00692 +/− 0.000084	0.00406	0.01595	0.255	1.18	0.122	2.22
*Recq4*	1878	0.00268	0.00273 +/− 0.000084	0.00144	0.00724	0.199	1.45	0.231	0.42
*Spo11-1*	948	0.00168	0.00097 +/− 0.000116	0.00043	0.00265	0.162	0.52	1.172	0.73
*Spo11-2*	984	0.00458	0.00444 +/− 0.000136	0.00232	0.01085	0.214	1.31	0.244	-^c^

^a^ *d*_N_/*d*_S_ was calculated relative to *Z. luxurians*, except for *Mus81*, where it was calculated relative to Z. *diploperennis*

^b^ The K scale factor describes sequence divergence by measuring the overall size of the phylogenetic tree [107]. Smaller K scale factor values indicate more divergence. We calculated the K scale factors separately for each of the two copies of *Mre11* and *Rad51* even though maize is the only of the species used in this analysis that has two homologs of these genes. The values differ somewhat for each of the two *Mre11* and *Rad51* homologs, which could suggest that the two copies are fairly diverged. However, according to genetic studies (see Discussion) the two copies of *Rad51* and *Mre11* have overlapping functions, which indicates that they can both be treated as functional homologs of the Rad51 and Mre11 genes from other species

^c^ The eukaryote-wide rate of sequence divergence could not be calculated as *Spo11-2* forms a separate lineage in plants and is absent from other extant groups of eukaryotes

We also investigated sequence diversity at the amino acid level. To do this, we calculated the ratio of nucleotide diversity at non-synonymous vs. synonymous sites (*π*_*a*_/*π*_*s*_) and the number of polymorphic amino acid residues per 100 residues. Here, we also observed substantial differences among the genes, although the patterns were different from those of nucleotide diversity (Table 1).

In addition to studying the coding regions, we examined the 5′ and intron regions of the genes (Additional file 1: Figure S2). As expected, non-coding regions exhibited higher levels of sequence diversity than coding regions. We anticipated observing the highest level of sequence diversity in introns but found that in some genes the level of sequence polymorphism was higher in the 5′ regions than in introns. The average *π* value for the whole sequenced length of the eleven recombination genes was 0.0026, which is 60% lower than the maize genic site average of 0.0067 [23].

Overall, the analyses we performed revealed that different genes exhibit distinct sequence diversity patterns, rather than showing similar evolution trajectories, as generally exhibited by domestication genes. Some recombination genes in maize, including *Rad51A1* and *Spo11-1*, showed low diversity in both DNA and amino acid sequences. In contrast, *Rad51A2* had relatively high DNA as well as amino acid sequence diversity. In yet another group of genes, which included *Mre11B* and *Mus81-1*, the diversity at the nucleotide sequence level was not related to the diversity at the amino acid sequence level.

### Sequence diversity in *Z. mays* ssp. *parviglumis*, tropical, and temperate maize inbreds

**Fig. 3:**
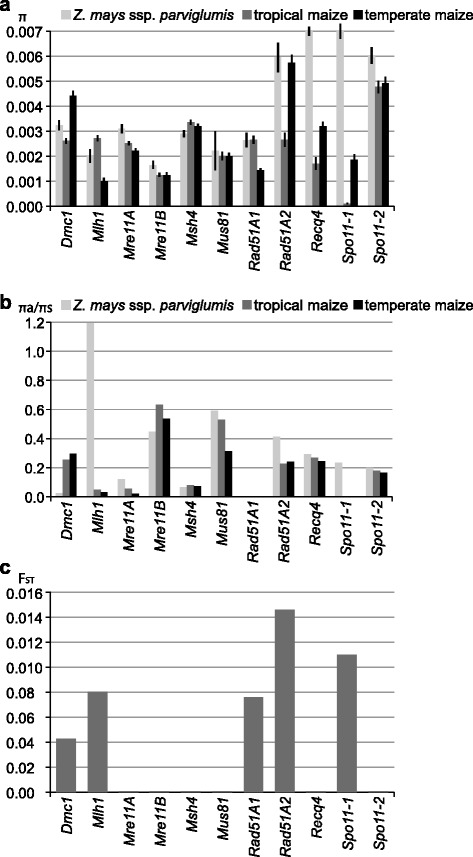
Comparison of sequence diversity rates in meiotic recombination genes in *Z. mays* ssp. *parviglumis* and in tropical and temperate maize inbreds. **a** Nucleotide sequence diversity (*π*) in the coding region. *Black bars* indicate standard error values. **b** Amino acid sequence diversity (*π*_*a*_/*π*_*s*_). In *Spo11-1*, all substitutions within the tropical as well as temperate inbred groups were non-synonymous and the *π*_*a*_/*π*_*s*_ ratios could not be calculated for them. **c** Fixation index (F_ST_) to measure genetic differentiation between temperate and tropical maize in coding regions of recombination genes

Following domestication, maize has adapted to a number of distinct environmental conditions after domestication and is now grown in many parts of the world. To gain insight into whether germplasms grown in different environments have accumulated different polymorphisms in meiotic recombination genes, we examined diversity rates separately in temperate and tropical maize inbreds. We also included in the analysis accessions of *Z. mays* ssp. *parviglumis*. Following its initial domestication from *parviglumis* and cultivation in Central America, maize was brought to North America about 4000 years ago [67]. This migration resulted in a split into tropical and temperate germplasms. The set of 31 diverse maize inbreds used in our study contained 14 temperate and 17 tropical lines. To compare the three germplasm pools, we computed the *π* statistic to examine DNA sequence diversity of the genes’ coding regions and the *π*_*a*_/*π*_*s*_ ratio to examine amino acid sequence diversity. Tropical maize is known to harbor higher overall genetic diversity than temperate maize [63, 68]. However, in the recombination genes, we observed a somewhat lower average *π* value (by about 15%) in the tropical inbred set than in the temperate set (Fig. 3a). Several genes, *Dmc1*, *Rad51A2*, *Recq4*, and *Spo11-1*, showed *π* values that were substantially lower in tropical germplasm than in temperate germplasm. On the other hand, tropical inbreds showed a higher average *π*_*a*_/*π*_*s*_ ratio (Fig. 3b).

To further examine differences between temperate and tropical maize, we calculated the fixation index (F_ST_), which is a measure of population differentiation based on genetic structure [69]. F_ST_ value of one indicates that two populations do not share any genetic diversity, while low F_ST_ values suggest lack of genetic differentiation. Of the eleven genes, only *Rad51A2* exhibited substantial differentiation between temperate and tropical inbreds (Fig. 3c).

Altogether, the data suggested that several recombination genes exhibited differences in sequence polymorphisms between temperate and tropical inbreds. However, only in *Rad51A2* these differences were large enough to suggest clear differentiation between temperate and tropical maize.

### Patterns of sequence divergence in recombination genes

A simple explanation for different recombination genes exhibiting different levels of sequence diversity may be that some recombination proteins exhibit fewer functional constraints and evolve faster than others. To investigate if this was indeed the reason behind our findings, we examined how fast different recombination genes evolve. To do this, we used two measures of sequence divergence. First, we calculated *d*_N_/*d*_S_, the ratio of the number of non-synonymous substitutions per non-synonymous site (*d*_N_) to the number of synonymous substitutions per synonymous site (*d*_S_) relative to a closely related sister species, which is a measure of sequence change over a fairly short evolutionary time (Table 1). As an outgroup, we used *Z. luxurians* for all genes except *Mus81-1*, in which we used Z. *diploperennis* because we could not obtain a sufficiently long sequence fragment for *Z. luxurians Mus81-1*. We detected variation in the *d*_N_/*d*_S_ ratios among the eleven recombination genes (Table 1). However, except *Spo11-1*, the ratios were relatively low (Table 1), which is consistent with presence of purifying selection. The higher *d*_N_/*d*_S_ ratio for *Spo11-1*, indicative of directional selection, may be a reflection of the fact that eukaryotic SPO11 proteins exhibit sequence conservation mainly in several domains and motifs, while the rest of the protein sequence shows higher levels of divergence [55, 70, 71].

To gain a measure of how fast recombination proteins diverge across longer evolutionary time, we calculated K scale factors using several fairly distant taxa (Table 1). This analysis compared the overall sizes of phylogenetic trees for each recombination protein to an arbitrary reference tree. The K scale factor is a measurement of the overall size of a phylogenetic tree in comparison to an arbitrary reference tree. K scale factors are smaller for proteins diverging faster than the reference, while proteins that diverge slower than the reference exhibit larger K scale factors. Core recombination proteins are considered to be generally very conserved but we found that the proteins in our study differed among each other in the K scale factor values by more than five-fold (Table 1).

Altogether, the *d*_N_/*d*_S_ and K scale factor analyses indicated that there were substantial differences in how fast different recombination genes evolved. *Rad51A1* and *Dmc1* exhibited the lowest levels of divergence while *Mlh1*, *Recq4*, *Spo11-1*, and *Mus81-1* exhibited the highest divergence levels among the 11 genes. However, differences in divergence rates among the different genes were not good predictors of differences in sequence diversity within maize. Out of the five genes with above-average divergence rates (*Mlh1*, *Mre11B*, *Mus81-1*, *Recq4*, and *Spo11-1*) only *Mlh1* and *Mre11B* exhibited above-average amino acid sequence diversity within maize. On the other hand, *Rad51A2*, one of the genes with low divergence rates, showed above-average diversity within maize.

### Selection patterns in recombination genes in maize and teosinte

To gain better understanding of molecular evolution patterns of recombination genes, we examined protein coding regions of the eleven recombination genes for signatures of selection. Selection in regulatory gene regions is thought to be associated with adaptive changes during maize domestication in many classes of genes, for example those controlling plant architecture, such as *teosinte branched 1* (*tb1*) [72]. However, expression differences are unlikely to underlie evolution of meiotic recombination genes, since there is little evidence of tight transcriptional regulation of recombination genes in plants. Even genes that are tightly regulated in other taxa, such as *Spo11* or *Hop2*, are ubiquitously expressed in plants [29, 73, 74].

To study selection patterns, we first examined the distribution of alleles in the population employing the commonly used frequency spectrum-based statistics Tajima’s D [75], and Fu and Li’s D and F [76] (Table 2). The three tests are based on a similar principle but are not identical and often provide complementary results. Tajima’s D is calculated using a difference between the mean number of pairwise differences sequences and the number of segregating sites. The Fu and Li’s D and F tests are based on similar algorithms but take into account the polarity of nucleotide changes relative to an outgroup. We used *Z. luxurians* as outgroup for all genes, except *Mus81-1*, for which we used *Z. diploperennis*. Tajima’s D and Fu and Li’s D and F tests found significant departures from neutral evolution in several maize genes. *Dmc1*, *Msh4*, *Rad51A2*, and *Spo11-2*, exhibited significant positive values of at least one of the tests, indicating that they could be under balancing (diversifying) selection [75, 76]. *Mlh1* and *Recq4* were marginally significant (*P* < 0.10). In contrast, *Mre11B* exhibited significant negative values, suggesting history of a selective sweep [75, 76].

**Table 2:** Results of frequency spectrum-based tests to detect selection patterns in coding regions of recombination genes in maize

Gene	Length (bp)	N	Fu & Li D		Fu & Li F		Tajima’s D		
			Value	Percentile of CS-generated distribution^a^	Value	Percentile of CS-generated distribution^a^	Value	Percentile of	
								empirical distribution^b^	CS-generated distribution^a^
*Dmc1*	1035	30	1.73842	94.0	1.8524	84.3	−0.50340	44.4	14.2
*Mlh1*	1589	26	−1.98286	1.3	−2.14871	1.5	−1.36469	4.7	3.7
*Mre11A*	2121	29	−0.78960	7.1	−0.86882	6.5	−0.57980	24.9	12.6
*Mre11B*	1792	31	−2.54110	0.1	−2.74082	0.4	−1.74966	6.4	1.5
*Msh4*	2415	31	1.82793	91.0	2.14002	87.5	1.65970	82.5	74.4
*Mus81-1*	1353	27	−0.45244	17.0	−0.51995	12.1	−0.39346	46.2	17.3
*Rad51A1*	1023	30	1.39229	91.5	1.22880	67.7	0.24754	38.1	33.3
*Rad51A2*	658	30	0.90418	40.0	1.73702	75.7	2.61602	99.9	96.5
*Recq4*	3528	22	1.54931	64.5	1.13756	42.5	−0.27752	10.2	18.9
*Spo11-1*	1158	30	0.70282	35.1	0.07890	18.3	−0.72935	27.5	10.0
*Spo11-2*	854	29	1.71266	90.0	1.38134	60.2	−0.01630	47.1	23.8

^a^ Percentile relative to a distribution of test values generated by coalescent simulations (CS) assuming neutral evolution and a domestication bottleneck

^b^ Percentile relative to the genome-wide distribution of Tajima’s D values from a survey of 703 random polymorphic loci in maize and *Z. mays ssp. parviglumis* by White et al. [22]. Tajima’s D values used in the comparison were calculated based only on the 14 inbreds used in the White et al. study [22]

For comparison, we conducted frequency spectrum-based tests for the *Z. mays* ssp. *parviglumis* accessions in the same manner as in maize. Here, we did not find significant departures from neutrality for any of the genes (Table 3). It should be noted, though, that the number of accessions that we used for *Z. mays* ssp. *parviglumis* was smaller than the number of accessions used in maize, which could have affected our results.

**Table 3:** Results of frequency spectrum-based tests to detect selection patterns in coding regions of recombination genes in *Z. mays ssp. parviglumis*

Gene	Length (bp)	N	Fu & Li D	Fu & Li F	Tajima’s D	
					Value	Percentile of empirical distribution^a^
*Dmc1*	1035	8	−0.72824	−0.70225	−0.16078	69.1
*Mlh1*	1589	6	0.03616	0.06771	0.12841	78.6
*Mre11A*	2121	6	1.22721	1.40326	0.97353	96.2
*Mre11B*	1792	8	−1.20536	−1.33713	−0.80674	37.9
*Msh4*	2415	8	−1.19426	−1.48169	−1.28039	17.2
*Mus81-1*	1353	NA	NA	NA	NA	NA
*Rad51A1*	1023	8	−1.68085	−1.83197	−1.06800	26.7
*Rad51A2*	658	7	0.77544	0.80139	0.33464	85.9
*Recq4*	3528	9	0.13432	0.02242	−0.27857	64.6
*Spo11-1*	1158	9	−1.28858	−1.38186	−0.72935	26.5
*Spo11-2*	854	9	−1.54205	−1.71638	−1.06063	26.7

None of the values indicate statistically significant departure from neutral evolution patterns

^a^ Percentile relative to the genome-wide distribution of Tajima’s D values from a survey of 703 random polymorphic loci in maize and *Z. mays ssp. parviglumis* [22]

Frequency spectrum statistics are known to be sensitive to demographic factors. A population bottleneck can result in strongly positive test values whereas a population expansion may cause negative values of the statistics [77]. To test the impact of selection vs. demographics, we used two tools to assess the statistical significance of the results of the three tests: (i) we compared Tajima’s D values for the eleven recombination genes to a genome-wide distribution of Tajima’s D values using an approach similar to the one employed to examine selection patterns in cell cycle genes in Arabidopsis [78], and (ii) we compared the values of the three statistics to critical values derived from coalescent simulations (CS) [79].

(i) The comparison of Tajima’s D values for specific loci to a genome-wide distribution of the statistic is based on a tenet that while selection acts on individual loci, demographics is likely to have a genome-wide effect. We compared Tajima’s D values for the recombination genes to a genome-wide distribution of Tajima’s D values based on a survey of 703 random polymorphic maize loci by Wright et al. [22]. This survey was conducted on a set of 14 maize inbreds, all of which were included in our inbred set. To conduct the comparison, we recalculated Tajima’s D values using only a subset of the 14 lines that were in common with the Wright et al. [22] study. Loci that showed no polymorphism in the Wright et al. [22] study were excluded from the dataset, as Tajima’s D statistic cannot be calculated for them. Tajima’s D values for *Mre11B* and *Rad51A2* fell into the extreme 2.5% fractions of the genome-wide Tajima’s D distribution (Table 2), indicating that they are significant at *P* < 0.05. As we did not have a genome-wide outgroup sequence data we could not make a similar comparison for the Fu and Li’s D and F tests.
(ii) To further evaluate the statistical significance of the Tajima’s D, and Fu and Li’s D and F statistics, we examined the probability of obtaining our empirical values under conditions of neutral evolution in coalescent simulations using Hudson’s ms program [80]. The simulations incorporated a population bottleneck under the parameters proposed for the maize domestication bottleneck by Wright et al. [22]. These analyses (Table 2) showed that the empirical Tajima’s D values for *Mre11B* fell into the low extreme 2.5% of simulated Tajima’s D values, indicating that they were significant at *P* < 0.05. *Rad51A2* was marginally significant (*P* < 0.07). Computation of CS-derived critical values for the Fu and Li’s F and D tests indicated that the empirical values for maize *Mlh1* and *Mre11B* were unlikely to result from neutral evolution alone, even under a domestication bottleneck affecting population demographics (Table 2).

As a complementary approach to investigating signatures of selection, we also used the likelihood ratio test (LRT) [81], which is based on a very different principle than the frequency spectrum tests. LRT examines ω, the ratio of the non-synonymous substitution rate *d*_N_ to the synonymous substitution rate *d*_S_ in gene coding regions. This method allows different ω values for individual codons, as different regions in the protein sequence may be under very different selection pressures and constraints, and is highly sensitive in detecting adaptive selection signatures. LRT can be used for within-species comparisons as long as sequence diversity is high and the level of intragenic recombination is low [82]. Therefore, prior to the analyses, we examined the data set for presence of intragenic recombination using the Genetic Algorithm for Recombination Detection (GARD) method [83]. In all of the eleven genes, we found no or low levels of recombination frequencies that did not exceed the rates acceptable for LRT analyses [82]. To further ensure that recombination did not affect the results of the test, for the genes where GARD detected recombination breakpoints, we individually tested the fragments separated by the recombination breakpoints. In each case, the results of the LRT analysis were identical to the results obtained using the entire gene coding region. For the LRT analysis, we only used lines in which the entire coding region of the gene was available. Overall, we found that coding regions of five genes, *Mlh1*, *Mre11B*, *Mus81-1, Rad51A2,* and *Spo11-2*, showed statistically significant signatures of positive selection (Table 4).

**Table 4:** Selection patterns in recombination genes in maize detected using the likelihood ratio test

Gene	Selection models^a^			% of codons		
	M0 vs. M3 (2Δ*l*) ^b^	M1 vs. M2 (2Δ*l*) ^c^	M7 vs. M8 (2Δ*l*) ^d^	Under purifying selection	Under positive selection	Evolving neutrally
*Dmc1*	15.32**	4.12	6.14	100	-	-
*Mlh1*	50.48**	19.46**	20.54**	93.3	6.7	-
*Mre11A*	1.06	0.46	0.12	100.0	-	-
*Mre11B*	24.64**	26.46**	14.23**	96.5	3.5	-
*Msh4*	0.00	0.00	0.00	100.0	-	-
*Mus81-1*	22.64**	15.62**	15.74**	97.3	2.7	-
*Rad51A1*	0.00	0.00	0.00	100.0	-	-
*Rad51A2*	43.76**	18.94**	20.62**	98.7	1.3	-
*Recq4*	0.00	0.00	0.00	100.0	-	-
*Spo11-1*	0.00	0.00	0.00	100.0	-	-
*Spo11-2*	80.52**	33.78**	35.18**	98.2	1.8	-

^a^ Likelihood ratio tests of selection models. Ratios statistically significant at *P =* 0.01 are denoted with **

^b^ Model M0 (one ratio) assumes a single dN/dS ratio across all sites in the gene’s coding region. Model M3 (discrete) assumes discrete classes of sites with different *ω* values. df = 4

^c^ Model M1 (neutral model) assumes all sites are either under purifying selection (ω < 0) or evolving neutrally (ω = 1). Model M2 adds a third category of sites under positive selection (ω > 1). df = 2

^d^ Model M7 (beta model) assumes that ω ranges from 0 (strong negative selection) to 1 (neutral evolution) and varies among sites in the gene’s coding region according to the beta distribution. Model M8 (beta& ω) similarly to M7 assumes that ω varies among sites but allows that, in addition to ranging from 0 to 1, ω may take values > 1 (positive selection). df = 2

### Presence of different selection patterns among maize inbreds

Because tropical and temperate maize inbreds differed in levels of sequence diversity in several recombination genes, we examined the patterns of selection separately in the tropical and temperate inbred pools using frequency spectrum tests. In *Mlh1*, *Mre11B*, and *Mus81-1* we found statistically significant differences in frequency spectrum test results between tropical and temperate inbreds (Table 5). *Mre11B* and *Mus81-1* showed departures from neutral evolution in the tropical inbred pool but not in the temperate pools whereas *Mlh1* exhibited the opposite trend.

**Table 5:** Analysis of selection patterns in coding regions of recombination genes in tropical versus temperate maize inbreds

Gene	Length (bp)	Population	N	Fu & Li D		Fu & Li F		Tajima’s D	
				Value	Percentile of CS-generated distribution^a^	Value	Percentile of CS-generated distribution^a^	Value	Percentile of CS-generated distribution^a^
*Dmc1*	1035	Tropical	16	0.63222	39.0	0.28466	29.3	−0.70093	14.4
		Temperate	14	1.31053	74.0	1.17209	59.4	0.15556	37.0
*Mlh1*	1589	Tropical	15	−0.73332	10.5	−0.79527	11.8	−0.47956	19.2
		Temperate	11	−1.73817	2.5	−2.12577	1.8	−1.87333	1.7
*Mre11A*	2121	Tropical	15	−1.42413	4.1	−1.40311	5.6	−0.54444	17.6
		Temperate	14	−0.83215	9.8	−0.70233	13.8	0.01684	31.9
*Mre11B*	1792	Tropical	17	−2.52246	0.4	−2.82179	0.3	−1.91094	1.6
		Temperate	14	−0.92817	9.1	−1.18564	8.4	−1.13145	8.8
*Msh4*	2415	Tropical	17	1.52595	76.6	1.73673	78.7	1.21920	69.6
		Temperate	14	0.37084	32.7	0.66771	38.1	0.98108	63.7
*Mus81-1*	1353	Tropical	13	−1.76372	2.5	−1.93612	2.8	−1.21317	7.0
		Temperate	14	0.78058	51.0	0.96432	55.9	0.87860	63.6
*Rad51A1*	1023	Tropical	16	1.46511	94.0	1.40765	80.6	0.46965	49.6
		Temperate	14	1.07546	70.0	1.46973	84.0	1.75287	90.0
*Rad51A2*	658	Tropical	17	1.46511	77.3	1.40765	66.2	1.21340	69.8
		Temperate	13	1.54333	83.0	1.72919	83.0	1.18039	71.9
*Recq4*	3528	Tropical	11	−1.30459	4.8	−1.65134	4.2	−1.47246	6.2
		Temperate	11	0.56264	41.1	0.48653	37.9	0.00246	34.2
*Spo11-1*	1158	Tropical	16	−1.52257	3.8	−1.65732	4.0	−1.16221	7.1
		Temperate	14	1.43044	80.0	1.19772	61.1	−0.09775	30.0
*Spo11-2*	854	Tropical	17	0.36324	29.8	0.38731	29.4	0.22491	37.5
		Temperate	12	−0.50395	16.0	−0.57496	16.8	−0.40399	21.2

^a^ Percentile relative to a distribution of test values generated by coalescent simulations (CS) assuming neutral evolution and a domestication bottleneck

To further explore differences in evolution patterns among inbreds, we investigated whether different gene genealogy branches exhibited different *d*_N_/*d*_S_ ratios. To conduct these analyses, we used LRT [81] to test whether any branches exhibit *d*_N_/*d*_S_ values statistically different from those of other branches in the genealogy [84]. We examined five genes, *Mlh1*, *Mre11B*, *Mus81-1*, *Rad51A2*, and *Spo11-1*, in which the overall *d*_N_/*d*_S_ ratios indicated presence of positive selection. We found that for three of the genes, *Mlh1*, *Mre11B*, *Mus81-1*, a model with two ratios, each for different branches, was marginally statistically significant (0.05 < *P* < 0.1) compared to a one-ratio model. The branches showing elevated *d*_N_/*d*_S_ ratios were those leading to CML52, CML69, CML333, CO255, and Ki11 for *Mlh1*, CML277 for *Mre11B*, and A344, CO255, Oh43, and P39 for *Mus81-1*.

## Discussion

### Polymorphism and selection patterns in recombination genes in maize and teosintes

Even though most core recombination proteins exhibit high degree of sequence conservation across eukaryotes, our analyses revealed fairly substantial levels of diversity and a variety of selection patterns. Using two very different approaches to identify sequence patterns indicative of selection, LRT and frequency spectrum tests, we identified a largely overlapping set of genes as possible selection targets. Frequency spectrum tests revealed patterns indicative of selection in coding regions of four genes, *Mlh1*, *Mre11B*, *Mus81-1*, and *Rad51A2*. The four genes, along with *Spo11-2*, were also identified by LRT as subject to positive selection. These observations were further corroborated by analyses of sequence diversity and divergence patterns. *Mlh1*, *Mre11B*, and *Mus81-1* displayed some of the highest sequence divergence rates among the recombination genes that we analyzed, which is consistent with them being subject to directional selection specifically in the maize lineage. *Rad51A2* showed low sequence divergence, which is typical of *Rad51A* homologs, but its diversity rates in maize were above the average for the eleven genes, which is consistent with the gene being subject to diversifying selection. Because we examined a group of genes, results of a single test alone may not provide very strong evidence for a particular gene to be a selection target because of multiple testing. However, consistently identifying the same genes in different tests provides stronger evidence for them indeed experiencing selection pressure.

Presence of a selection signature at a specific locus using frequency spectrum tests may imply that the locus itself is a target of selection but it also may be caused by proximity to another locus that is a strong selection target (hitchhiking effect) [85]. However, in maize, linkage disequilibrium (LD) decays quite rapidly [68] and the gene density is quite low, raging from 9 to 200 kbp per gene. Consequently, the hitchhiking effect is unlikely to be a major source of non-neutral evolution patterns. Furthermore, LRT, which produced very similar results to those of the frequency spectrum tests, is not sensitive to the hitchhiking effect [81].

To further explore the possibility of hitchhiking, we examined the locations of the 11 recombination genes with regard to regions of selective sweep previously identified in the maize genome [86, 87]. We found that *Mre11B* was in a known selective sweep region [87], while the other ten genes were 100 kbp away (*RAD51A1*) or more from regions of selective sweep. More detailed analyses of the sweep region encompassing *Mre11B* showed that *Mre11B* was the only *bona fide* gene located within the region, whereas all other ORFs represented transposons or low-confidence genes. Consequently, we believe that *Mre11B* may be the target of the selective sweep.

Previous analyses of genome-wide diversity patterns in maize have shown that a relatively small number of maize genes, about 2–4%, have experienced extremely strong selective sweeps during maize domestication [19, 20, 22]. These sweeps led to very severe diversity losses at the affected loci and their targets were mostly genes controlling plant architecture and critical agronomic traits (“domestication traits”) [22, 72]. The recombination genes examined in this study do not appear to be in the same category of selection targets and exhibit higher levels of polymorphism in maize than the domestication genes regulating agronomic and plant architecture traits. Consequently, the selective pressures experienced by the recombination genes are likely to be lower than those experienced by the domestication genes.

The predominant type of selection we uncovered was diversifying selection, suggesting that selection pressures experienced by recombination genes vary among maize lineages. This observation is consistent with the finding of differences in evolution patterns between the tropical and temperate inbred sets. It is conceivable that selection pressure affecting recombination genes is episodic. In some situations, increased meiotic recombination rates may be favored, as they facilitate formation of new gene combinations. In other lineages, lower recombination rates may help preserve linkage blocks of advantageous alleles. The fact that QTLs for recombination frequencies are not widely shared among diverse maize inbreds [64], although they are detected in individual mapping populations [88], provides additional credibility to this claim. Future analyses of larger sets of maize inbreds may help discern whether lineage-specific selection patterns in recombination genes indeed exist.

### Potential functional consequences of sequence polymorphisms

**Fig. 4:**
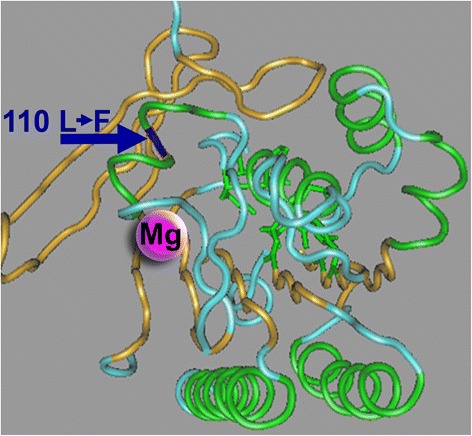
Flat projection of the three-dimensional structure of the BRC repeat in the RAD51A protein. The position corresponding to the polymorphic residue 110 in maize RAD51A2 is marked with an *arrow*. This amino acid is located at the base of a small loop on the protein surface that is adjacent to the Mg^2+^-binding pocket in the ATPase domain

One of the tests of adaptive evolution is identifying functional differences between polymorphic alleles of the gene. However, this kind of analysis is not currently feasible for recombination pathway components. Nevertheless, numerous functional domains have been identified in recombination proteins and their three-dimensional structures have been elucidated. To investigate if any of the amino acid polymorphisms that we identified could be associated with functional changes in the proteins, we examined the positions of the polymorphic residues in predicted three-dimensional proteins structures. These analyses identified several polymorphisms that have the potential to considerably affect protein structure. One of notable polymorphisms was an alanine to phenylalanine change in RAD51A2 at residue 110, which is located in a conserved ATP-binding site of the protein. We found that this residue flanks a small loop on the protein surface that is adjacent to the Mg^2+^-binding pocket in the ATPase domain (Fig. 4). Mg^2+^ binding is known to induce a conformational change in the RAD51 protein, which is required for DNA binding [89]. Alanine is present at this residue in all eukaryotes that we examined, except for the two unusual RAD51A proteins in *Physcomitrella* [90], which instead contain phenylalanine. Another example of a polymorphism with a potential to result in a functional change was a substitution of highly conserved hydrophilic serine by hydrophobic glycine at position 330 in SPO11-2. This residue is located on the surface of the TOPRIM domain in the SPO11 protein, on the face of the protein that directly interacts with the DNA [91]. Overall, sequence polymorphisms in recombination genes could, for example, be associated with increased meiotic recombination rates. They could also affect the distribution of crossovers across the genome or affect the frequency of ectopic recombination between repetitive or homeologous genome regions.

Several proteins involved in the meiotic recombination pathway, including MLH1, MRE11, MUS81, RAD51, and RECQ4, also function in somatic DNA repair. However, we did not find obvious differences between the evolution patterns of genes encoding these proteins and genes encoding proteins with exclusively meiotic functions, such as Dmc1, Msh4, and Spo11. It could be speculated that perhaps the meiotic functions are predominant in the dual-function genes or that meiotic functions are the ones that predominantly experienced selection pressures during maize evolution. Mutants in most meiotic recombination genes in plants do not show somatic defects, unless artificially exposed to genotoxic stress [43, 45, 52, 92, 93], suggesting that these genes are not absolutely required for somatic growth and development. However, growth conditions in controlled experimental environments may not mimic the conditions that plans encounter in their natural environments and evolution of somatic DNA repair functions of recombination genes cannot be categorically excluded. For example, maize could have experienced changing levels of genotoxic stress, such as UV radiation during its geographic diversification. Finally, many recombination proteins act as components of large protein complexes, and changes in some proteins could induce co-evolutionary changes in proteins that interact with them.

### Duplicated recombination genes

Three of the recombination genes examined in this study, *Mre11*, *Rad51*, and *Spo11*, are present in the maize genome as duplicated copies. Two duplications (*Rad51* and *Mre11*) likely took place at the base of the grass lineage and one (*Spo11*) in the ancestor of all extant eukaryotes. Interestingly, we did not find any duplications that would trace their origin to the allopolyploidization event that took place in the direct ancestry of maize [94]. This observation is consistent with previous predictions, based on analyses of gene duplications in Arabidopsis, that DNA metabolism genes are preferentially subject to gene loss following whole-genome duplication [95].

We observed substantial differences in evolution patterns between the *Mre11*, *Rad51A*, and *Spo11* paralogs. In all three cases, the paralogs exhibited large differences in the levels of sequence diversity and divergence. We should note, however, that even though we see commonalities in the behavior of the three duplicated gene pairs, the mechanisms of their evolution may not necessarily be the same because of the extreme difference in the ages of these duplication events (i.e. the *Spo11* duplication being much more ancient that the *Rad51* and *Mre11* duplications).

Overall, our data suggested that the paralogs in the three pairs of duplicated maize recombination genes have acquired distinct functions, which is known to follow gene duplications [96], and are under differing selection pressures and constraints. Although the functions of the two *Spo11* genes have not been studied in maize yet, in Arabidopsis both *Spo11-1* and *Spo11-2* are required for proper progression of recombination, which is consistent with each of the genes having a somewhat distinct function. In contrast, the two *Rad51A* genes in maize were found to be redundant in mutant analyses [49]. Presence of different selection patterns in *Rad51A1* and *Rad51A2* suggests that differences in the functions of the two genes are likely to exist after all. Interestingly, a recent study in rice found that although both RAD51A1 and RAD51A2 can bind ssDNA and dsDNA, RAD51A2 exhibits much higher strand exchange activity than RAD51A1, indicating that the two proteins are functionally different [97].

## Conclusions

Analysis of eleven genes controlling key steps in the meiotic recombination pathway in a diverse set of maize and teosinte lines uncovered substantial levels of sequence polymorphism in most of the genes and identified signatures of adaptive evolution in several of them. The data show that despite their ancient origin and overall conservation, recombination genes can exhibit extensive and complex patterns of molecular evolution. At least some of the sequence polymorphisms that we found have the potential to cause changes in the functioning of the recombination pathway. Such changes could have contributed to the successful domestication of maize and its subsequent expansion to new cultivation areas.

Six of the eleven recombination genes represented ancient gene duplications events. We found that the duplicated genes exhibited distinct patterns of sequence diversity even in the case of duplicates that appear to be functionally redundant in genetic experiments. These results show that evolutionary analyses are useful in complementing genetic analyses when studying functions of duplicated genes.

## Methods

### Plant material

Sequence diversity in recombination pathway genes was examined in 31 maize inbreds and 14 teosinte accessions. The maize inbreds were: A188, A344, B73, B97, CML52, CML69, CML103, CML228, CML247, CML277, CML322, CML333, CO106, CO125, CO255, HP301, Il14h, Ki3, Ki11, Ky21, M37W, M162w, Mo17, Mo18w, MS71, NC350, NC358, Oh7b, Oh43, P39, and Tx303). The teosinte accessions included nine *Z. mays* ssp. *parviglumis* lines, eight of which came from a set developed by John Doebley (University of Wisconsin, Madison) (TIL01, TIL02, TIL05, TIL07, TIL11, TIL15, TIL16, and TIL17), and one from the CIMMYT collection (TL74A J2 K67-5). The other teosinte lines were *Z. mays* ssp. *mexicana* (K69-7 and BA93 WST 85-2), *Z. mays* ssp. *huehuetenangensis* (TL93B Teo Huehue), *Z. diploperennis* (JAL87 Las Joyas), and *Z. luxurians* (TL92B TEO-Guate). Seeds and/or tissue samples for all lines except A188, A344, and Mo17 were kindly provided by Ed Buckler (USDA-ARS and Cornell University, Ithaca, NY).

### Gene sequences

Genomic regions of maize recombination genes were identified in the whole-genome sequence of the maize B73 inbred [50] using sequences of known Arabidopsis and maize recombination genes as BLAST queries. Gene coding regions were delineated using full-length cDNA and EST sequences available in the GenBank. For *Mlh1*, *Msh4*, *Mus81*, and *Recq4*, only partial EST sequences were present in the GenBank. We determined the full coding regions of these genes using RT-PCR, which was performed as previously described [98].

In addition to full-length gene copies, we discovered in the maize genome truncated fragments of *Dmc1*, *Mlh1*, and *Spo11-2* (Additional file 1: Table S1). These partial gene copies represented different fragments of the corresponding genes, always included several exons and introns, and exhibited nearly 100% sequence identity to the corresponding regions of their full-length relatives. They were flanked by DNA showing no similarities to the full-length genes. These observations suggested that the truncated copies of *Dmc1*, *Mlh1*, and *Spo11-2* are relatively recent pseudogenes.

To obtain sequences of the eleven recombination genes from the set of maize inbreds and teosinte lines for sequence polymorphism analyses, PCR primers (Additional file 1: Table S3) were designed to amplify full-length genomic regions of each gene. Nearly all primers were designed to anneal in introns to obtain entire coding regions and ensure that only orthologous sequences were amplified. When selecting primer sites, we avoided regions present in multiple copies in the maize genome sequence.

Sequencing was performed directly on PCR products in both orientations with BigDye v3.1 (Applied Biosystems, Foster City, CA), and analyzed using the Applied Biosystems 3730 automated sequence analyzer. Manual sequence editing was conducted using Sequencher (Gene Codes Corp., Ann Arbor, MI).

### Phylogenetic analyses

Alignments of protein sequences from several species of eukaryotes were performed using ClustalX [99] and adjusted manually. Alignment gaps were excluded from analyses. Maximum parsimony analyses of protein sequences were conducted using PAUP 4.0 [100]. Bayesian analyses were performed using MrBayes 3.1.2 [101] using the Poisson model [102]. TreeviewPPC [103] was used to display phylogenetic trees. To compare evolution rates between different branches of phylogenetic trees, we used the Tajima’s 1D relative rate test [104] implemented in MEGA4 [105].

### Sequence divergence and diversity analyses

Nucleotide diversity measures: *π* [65] and *θ*_*W*_ [66] were calculated using DNAsp v.5 [106]. K tree analysis was used to examine the rates of divergence of recombination genes across eukaryotes [107]. One of the outcomes of this analysis is the K scale factor, which is a comparison of the overall sizes of two phylogenetic trees. We constructed Bayesian trees based on protein sequences of the recombination proteins and compared them to an arbitrarily selected reference tree constructed from concatenated sequences of all of the examined recombination proteins. To prevent the comparison from being confounded by differences in tree topology, we arbitrarily selected five fairly distantly related taxa, *S. cerevisiae*, human, Arabidopsis, rice, and the B73 inbred of maize. The K scale factor analysis was conducted with the Ktreedist_v1 program [107].

### Selection analyses

To examine DNA sequences for presence of selection signatures, we used the Tajima’s D [75], Fu and Li’s D [76], Fu and Li’s F [76] tests as implemented in DNAsp v.5 [106]. We used coalescent simulations to validate the results of these tests and examine whether deviations from neutral evolution may have been caused by demographic factors rather than selection. To conduct coalescent simulations, we utilized Hudson’s ms program [80]. We generated 10,000 coalescent simulations using previously described parameters [22]. A conservative assumption of no intra-locus recombination was used in the simulations [79]. The population mutation parameter *θ* was estimated from teosinte data. To simulate the domestication bottleneck, the value for the bottleneck severity parameter (*k*) was set for 2.45 [22]. This parameter is the ratio of population size during bottleneck to bottleneck duration. Critical values for neutrality tests were computed using the msstats software (https://github.com/molpopgen/msstats) modified by Eli Stahl.

We also used the likelihood ratio test (LRT) [81], which examines ratios of non-synonymous to synonymous nucleotide substitution rates and conducts pair-wise comparisons between several models that describe different selection patterns defined by the ratio of non-synonymous (*d*_N_) to synonymous (*d*_S_) substitution rates to identify the best-fitting model for each gene. These patterns include purifying selection (*d*_N_/*d*_S_ < 1), neutral evolution (*d*_N_/*d*_S_ = 1), and positive selection (*d*_N_/*d*_S_ > 1). The analysis was performed with the codeml program in the PAML package [108] using coding regions of the recombination genes. The LRT method is not reliable when recombination is frequent among the examined haplotypes [82]. Therefore, prior to the LRT analysis, we determined that recombination frequencies in the coding region of each gene did not exceed the acceptable limits [82] using the Genetic Algorithm for Recombination Detection (GARD) method [83] conducted using a web interface (http://www.datamonkey.org/dataupload.php). This method identifies recombination breakpoints in sequence alignments by searching for phylogenetic incongruence.

### Protein structure predictions

To determine the locations of polymorphic amino acid residues in three-dimensional protein structures, we conducted protein structure prediction analyses. Maize protein sequences were threaded using Cn3D (http://www.ncbi.nlm.nih.gov/Structure/CN3D/cn3d.shtml) on the available empirical three-dimensional structures of the MutL transducer domain (MMDB ID: 10447) in MLH1, the phosphoesterase domain (MMDB ID: 34451) in MRE11, the ERCC domain (MMDB ID: 52594) in MSH4, the ERCC domain (MMDB ID: 52594) in MUS81, the BRC repeat (MMDB ID: 21264) in RAD51, the DEXDc domain (MMDB ID: 13107), the HELICc domain (MMDB ID: 12961), the REQC domain (MMDB ID: 36694), and the HRDC domain (MMDB ID: 34680) in SGS1, and the TOPRIM domain (MMDB ID: 11634) in SPO11.

## Additional file

Additional file 1:
**Table S1.** Maize genes encoding key meiotic recombination proteins. **Table S2.** Accession numbers for sequences from GenBank used in the phylogenetic analysis of recombination genes. **Table S3.** PCR primers that were used to amplify sequences of meiotic recombination genes from maize. **Figure S1.** Phylogeny reconstructions of the DMC1, MLH1, MSH4, and RECQ4 proteins in eukaryotes based on the Bayesian and maximum parsimony methods. **Figure S2.** Comparison of nucleotide sequence diversity rates (π) in the promoter, coding, and intron regions of meiotic recombination genes in *Z. mays* ssp. *parviglumis* and in tropical and temperate maize hybrids. (PDF 264 kb)

## Abbreviations

CO: Crossover
CS: Coalescent simulation
*d*_N_: Number of non-synonymous substitutions per non-synonymous site
*d*_S_: Number of synonymous substitutions per synonymous site
DSB: Double-strand break
F_ST_: Fixation index
GARD: Genetic Algorithm for Recombination Detection
LRT: Likelihood ratio test
MP: Maximum parsimony
NCO: Non-crossovers
ssDNA: single-stranded DNA
*θ*_*W*_: Scaled measure of the number of polymorphic nucleotide sites per nucleotide
*π*: Nucleotide diversity; average number of nucleotide differences per site between any two sequences in sample
*π*_*a*_: Nucleotide diversity at non-synonymous sites
*π*_*s*_: Nucleotide diversity at synonymous sites
ω: *d*_N_/*d*_S_
