# Supplementary material for: Evolution of meiotic recombination genes in maize and teosinte
Source: BMC Genomics. 2017 Jan 25;18:106. doi: 10.1186/s12864-017-3486-z (PMC5267385; doi:10.1186/s12864-017-3486-z)
Supplement: Additional file 1: — Table S1. Maize genes encoding key meiotic recombination proteins. Table S2. Accession numbers for sequences from GenBank used in the phylogenetic analysis of recombination genes. Table S3. PCR primers that were used to amplify sequences of meiotic recombination genes from maize. Figure S1. Phylogeny reconstructions of the DMC1, MLH1, MSH4, and RECQ4 proteins in eukaryotes based on the Bayesian and maximum parsimony methods. Figure S2. Comparison of nucleotide sequence diversity rates (π) in the promoter, coding, and intron regions of meiotic recombination genes in Z. mays ssp. parviglumis and in tropical and temperate maize hybrids. (PDF 264 kb) [file 12864_2017_3486_MOESM1_ESM.pdf]

Table S1. Maize genes encoding key meiotic recombination proteins.

Gene	Coding sequence length	ORF length	Chromosome/ bin	Number of related pseudogenes[a]
Dmc1	1035bp	4059bp	3/09	12
Mlh1	2178bp	6869bp	8/06	4
Mre11A	2121bp	4867bp	2/02	-
Mre11B	2019bp	5389bp	4/04	-
Msh4	2415bp	51074bp	2/06	-
Mus81-1	1353bp	3409bp	3/05	-
Rad51A1	1023bp	3589bp	7/04	-
Rad51A2	1023bp	2815bp	3/05	-
Recq4	3528bp	12455bp	5/04	-
Spo11-1	1152bp	4316bp	5/01	-
Spo11-2	1149bp	>2513bp[b]	4/04	1

[a] The pseudogenes are truncated fragments of the full-length gene integrated in different genomic locations.

[b] Actual ORF length unknown because of a genome assembly gap in intron 7.

Table S2. Accession numbers for sequences from GenBank used in the phylogenetic analysis of recombination genes.

Protein	Species	GenBank source
DMC1	*Saccharomyces cerevisiae*	NP_011106.1
	Homo sapiens	NP_008999.2
	Arabidopsis thaliana	NP_188928
	Oryza sativa DMC1A	AAM76793.1
	Oryza sativa DMC1B	NP 001065738.1
	Zea mays	NP 001141379.1
MLH1	*Saccharomyces cerevisiae*	AAA16835.1
	Homo sapiens	NP 005582.1
	Physcomitrella patens 126125	XP 001763041.1
	Arabidopsis thaliana	NP 567345.2
	Oryza sativa	NP 001045457.1
MRE11	*Saccharomyces cerevisiae*	AAB61454.1
	Homo sapiens	NP 005582.1
	Physcomitrella patens	XP 001755538.1
	Arabidopsis thaliana	NP 200237.1
	Oryza sativa MRE11A	NP 001054012.1
	Oryza sativa MRE11B	BAD03045.1
	Zea mays MRE11A	ABQ59303.1
	Zea mays MRE11B	NP 001106049.1
MSH4	*Saccharomyces cerevisiae*	P40965
	Homo sapiens	AAB72039.1
	Physcomitrella patens 145361	XP 001777754.1
	Arabidopsis thaliana	NP 193469.2
	Oryza sativa	NM 001066195.1, conceptually spliced and edited
MUS81	*Saccharomyces cerevisiae*	NP 010674.1
	Homo sapiens	NP 079404.3
	Physcomitrella patens 176090	XP 001754940.1
	Arabidopsis thaliana	NP 194816.2
	Oryza sativa	NP 001045390.1
	Sorghum bicolor MUS81-1	sorghum genome sequence, conceptually spliced
	Sorghum bicolor MUS81-2	sorghum genome sequence, conceptually spliced
RAD51	*Saccharomyces cerevisiae*	NP 011021.1
	Homo sapiens	NP 002866.2
	Physcomitrella patens RAD51A	CAC86603.1
	Physcomitrella patens RAD51B	CAC86604.1
	Arabidopsis thaliana	NP 568402.1
	Oryza sativa RAD51A1	BAB85491.1

	Oryza sativa RAD51A2	NP 001066806.1
	Zea mays RAD51A1	NP 001104918.1
	Zea mays RAD51A2	NP 001104919.1
SGS1/	*Saccharomyces cerevisiae*	NP 013915.1
RECQ4	*Homo sapiens*	AAI07424.1
	Physcomitrella patens 132154	XP 001767449.1
	Physcomitrella patens 1048	XP 001778692.1
	Arabidopsis thaliana RECQ4A	NP 172562.2
	Arabidopsis thaliana RECQ4B	NP 176289.6
	Oryza sativa	AK242898.1
SPO11	*Sulfolobus acidocaldarius* TOP6A	YP 255943.1
	Giardia intestinalis	XP 001706588.1
	Saccharomyces cerevisiae	NP 011841.1
	Neurospora crassa	CAB88597.1
	Coprinus cinereus	AAF26720.1
	Schizosaccharomyces pombe	NP 593479.1
	Homo sapiens	NP 036576.1
	Mus musculus	NP 001077429.1
	Physcomitrella patens 126981	XP 001778952.1
	Physcomitrella patens 115757	XP 001763679.1
	Arabidopsis thaliana SPO11-1	NP 187923.1
	Arabidopsis thaliana SPO11-2	NP 176582.2
	Arabidopsis thaliana SPO11-3	NP 195902.1
	Oryza sativa SPO11-1	AAP68363.1
	Oryza sativa SPO11-2	NP 001061027.1
	Oryza sativa SPO11-3	BAF65346.1
	Oryza sativa SPO11-4	NP 001067307.1
	Sorghum bicolor SPO11-1	XP 002466440.1
	Sorghum bicolor SPO11-2	sorghum genome sequence, conceptually spliced
	Sorghum bicolor SPO11-3	XP 002468055.1
	Zea mays maize SPO11-1	EU969350.1, edited
	Zea mays maize SPO11-2	ACG46441.1
	Zea mays maize SPO11-3	NP 001141583.1

Table S3. PCR primers that were used to amplify sequences of meiotic recombination genes from maize and teosinte lines for polymorphism analyses.

Gene	Primer pair	Forward primer	Reverse primer
Dmc1	1	CTCAGCACCCTCAAGTACACAG	CACTAATGCGGGAGGAGAGA
	2	TAATCCACCTTTGCAGTTTTGA	AGTACCCTTGTCCTTCTGCGT
	3	AACACTTTGCATCACAGAGGC	GTCGGCTGGAAACTTCATTAA
	4	GGTTGCCTACATTGACACTGA	CCACTGAAATCAACACGGAAC
	5	GTTCCGTGTTGATTTCAGTGG	CCGAATCAGTCTTTTACGTCC
Mlh1	1	ACGGAGGGAGTAGTTGTTTTTATAT	CTCGACGCTAGAAAAGTGGG
	2	ATATTTTCGTGGTTTCACCTTAA	TATTCGTTGCCTAAAAAAATCACT
	3	CCGCTCCTTTTTGTTTTCTACT	ACCACGTAAGATCAATGTTCATAAA
	4	CTTTGATGTGAACTTCTGTTTCTG	AATAACCTCTAAAGACCTGCAGC
	5	TGCTGCAGGTCTTTAGAGGTTA	TATTTTCGAGTTTGCCTGCA
	6	TGCAGAGGTAAGTTATTTTTGGAA	AGGAGTGGTTTCAGGGTTGTC
	7	ATTGCTATTGGTGCTAAAAGGTT	TATAGGGTGTCTTTCTTGGCTTC
Mre11A	1	CTTCTCTTCCCTTGCTGCTG	TTTACACATTTTCACTAGTCCACAA
	2	TGCAGCAATGTTGTTCACTTTT	CAAAATGAAGGTCCAAAAACAA
	3	TTGTTTTTGGACCTTCATTTTG	TTTTTCCTTTCTTGATGATTTTTC
	4	GAAAAATCATCAAGAAAGGAAAAA	CAGGGAGCAAATGGACACAG
	5	GGGTTTGCACTCCTATACTTCTTT	CAGCCCCAGGAAAAATACAA
	6	TTGTATTTTTCCTGGGGCTG	TCGGCTCTTCCAACTTTACAA
Mre11B	1	TGAGGCTCGTTAATTTGTGTTC	GCCAGTCGCAAAATCAAAAC
	2	CACATTTTCATTCCTTTTGGG	TTAACAGAGAAACGTGAAGGAAAC
	3	AGCATTTTTTTGTACGTTCACTTAT	ACCTCTTGCCCTCTTTTTAAATA
	4	TGTTCTGTCTTTCCGCTTTTG	CCATATGAGCGTACCCTAGCA
	5	CATTCCATCTTTTTCTAGTATTGCT	TGGTGGTAGTTAATTTGTGATGC
	6	CCGCTGAAGAAAAAGTAGGATAT	CAACTAATCGATCAGGGTCCTC
Msh4	1	GGTCATTCCATTCCGTCACAT	TCCGACGAAATAACAGCTCAC
	2	TGATTTGGTCATCCTTTTTTGA	ACCGTAAAAGTTGAAATCCTGAA
	3	GTCATATCACCTTTTTTCTGGAAT	GAAACGACACGCAGGTATCC
	4	CTGGGAATTTGAGTTGTTGATCT	CTACGAGTAAAGACAATGGGACC
	5	CACAGGCCCAAGTCCATAAC	TTATGAAATTATGAAGGGAACCAA
	6	CTACGAGTTCCTGGTCATGCC	CACAGCGCTACGGATGACTAA
	7	TTGCTTTTTTGTTGATGTCCT	AGTTTTCGTGACCCTTCTCG
	8	GCTTTGAGTCTGCCATTTTTTA	TTTAGTCATTTTCAGCTCTTCTCG
	9	TTGCCATGGTTAGATATCAGAATTA	AAATGATCAGCAGTTGGGTACA
	10	CTAAAACCCCCCATTCTATTCA	AACATTTATTAAGGGCCAGAGC
Mus81-1	1	CCGCAAAATTCAAATCAGTTC	CAGACAGTTAAGCATGAAAATCG
	2	TTTTCCACACCAATTATCTTTTCT	GCAAGGTTCCTCATAATTTTAAGA
	3	TTGATATTTTTGTGTTGTCTTCCAG	GAAAAAGGTATTACTGTCGAGGC
	4	TGATCTCTTAGTTGTGCCATGTG	ATATGCCTCGAGATCACCAAGT
Rad51A1	1	TTGCCTATTGGTGTTGGTTGT	CAAGAAGCCGGTTAGGGTTA
	2	AGCTCCTATGGTTGCATTTTG	CCAACAGAACAATCAAGGAATG
	3	ATCCACCCTCTTTTTCCATTT	CACCTTTGATTCGTAAGCAGAA
	4	TCTGGCTGCTGTTCTGAATCT	CCAAGGAATGACAAAGCAAAT
Rad51A2	1	CAAAGCCAAAGTGAGGATGC	AACGGGACCTAAGATATTGGC
	2	TTGGGGAATTTTCATGTTAGAGA	GTGGTGCCATATCTTAGAATTTGT
	3	ACAAATTCTAAGATATGGCACCAC	AAGTAACGTTTGCATGAGGACA
	4	CTGCTATGAAATTACCGGACAA	ACGAGTGCAGCAGATCAGAGA
Recq4	1	CCAATCCCTTCTGTTCCCTT	AAGTCTCGACAAATGTGCTAAGC
	2	ATCCCTACCTTCTCTTTTACCTACA	TAGCCCCTCAGTATAGAACAAAGAT
	3	GGCTATTTCCGCATGTTATCT	AGCTGCTTAGAACCCATGACC

	4	TTTTCCTTTGCCAATCACTGA	TGTGGCGTGTCAGTAAAATAGAA
	5	CTGTTGAGACTGTTGGAAAATGTAT	GCTCCCAATCTATATCCAGGTAAT
	6	TGGCGTACAGATTTTGAACAGT	GTCCACGTCATTTTCACAGTAACT
	7	GCACTGAAGACACATAAGGACAAT	TGGAAACAAAAAATAAATAGAAACG
	8	CGTTTCTATTTATTTTTTGTTTCCA	AATACAATGACAGCCACACACAA
	9	CCCCCCCTCAAACTAACATAT	CTCATTTCCTTCAACGTGTCTG
	10	TTGTTGCACCTTGTCATTCG	AACCCTTGACATCACTGCATC
Spo11-1	1	TTGTTAATTTTTTGTCTTGAGAGGA	CCCTCAGGAGCACATCTACG
	2	CGTCCTCTCTCTGCTCCACA	AGCACACAGGATGATTAAGGAAC
	3	TTTGCTAGGTTTGCGATGTTATA	AAGAGCAAAAAATGATCAGTGACT
	4	GCATTATAGTGAGCTGAAGTTGTCT	GCATAGCAGCATCAGATAAATCA
	5	TGCTACTTGCGTAATTTCATTCT	ACTCTATGACACATCCTGGACG
Spo11-2	1	ACGCAGACCACTACTGGACTC	GAGGAAGTTGAGCACCGCTAC
	2	GTACACCAGTTGCCAAACCTC	ATAATTCCGTCCCAGGGTACT
	3	TATCCTCGCCCATTTGTCTG	ATGCTTGAAATGGAGGTACACA
	4	TTGCATCCAAATTCTTTATGATAA	TCGTCAGATGTAATCGCCCT

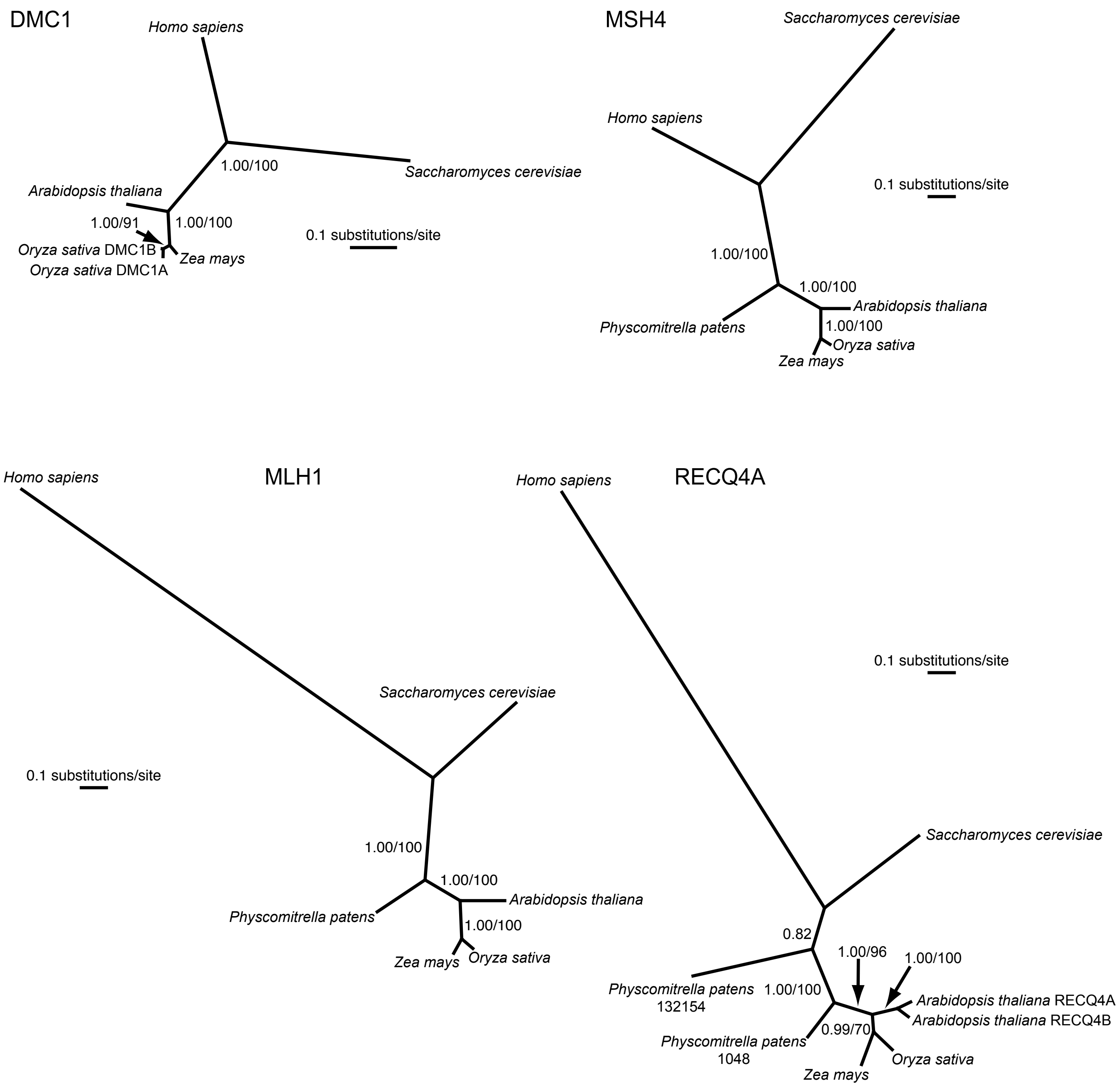

Figure S1. Phylogeny reconstructions of the DMC1, MLH1, MSH4, and RECQ4 proteins in eukaryotes based on the Bayesian and maximum parsimony methods. The Bayesian and the maximum parsimony trees were identical for all proteins. Numbers next to branches are posterior probabilities and bootstrap support values. Only posterior probability values of 0.8 and higher and bootstrap support values of 70% and higher are reported. Accession numbers for sequences from GenBank used in this analysis are listed in Table S2.

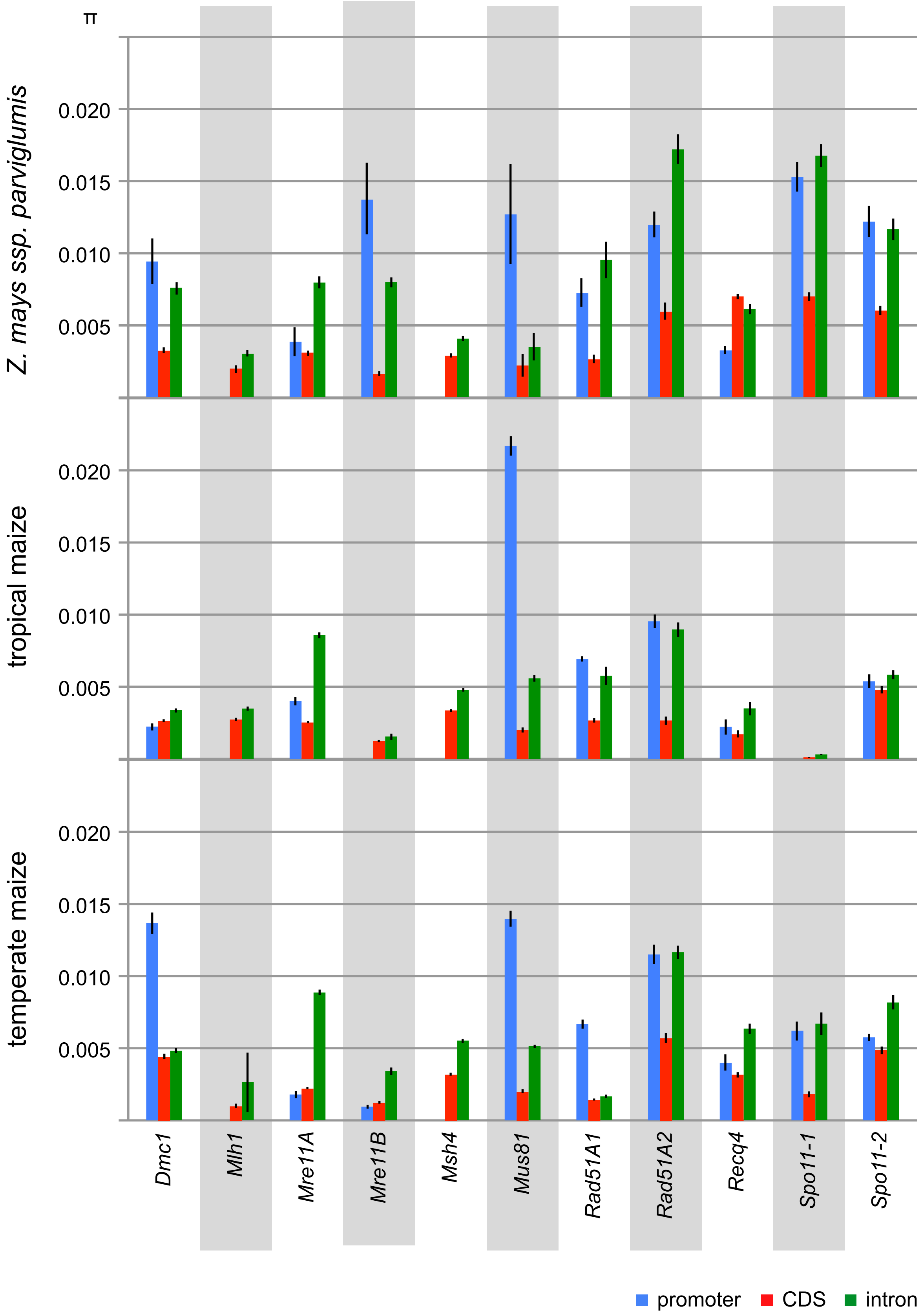

Figure S2. Comparison of nucleotide sequence diversity rates (π) in the promoter, coding, and intron regions of meiotic recombination genes in *Z. mays* ssp. *parviglumis* and in tropical and temperate maize hybrids. Black bars indicate standard error.
